# Characteristics of different asthma phenotypes associated with cough: a prospective, multicenter survey in China

**DOI:** 10.1186/s12931-022-02104-8

**Published:** 2022-09-12

**Authors:** Jianmeng Zhou, Fang Yi, Feng Wu, Pusheng Xu, Meihua Chen, Huahao Shen, Lin Lin, Yunhui Zhang, Suyun Li, Changgui Wu, Yadong Yuan, Gang Wang, Xianwei Ye, Ping Zhang, Huaping Tang, Qianli Ma, Lanqing Huang, Zhongmin Qiu, Haiyan Deng, Chen Qiu, Guochao Shi, Jiayu Pan, Wei Luo, Kian Fan Chung, Nanshan Zhong, Kefang Lai, the CPA Cohort Study Group, Ruchong Chen, Weijie Guan, Yanqing Xie, Mei Jiang, Jie Gao, Wen Hua, Guangyun Cai, Cuiyi Chen, Mingjuan Zhou, Yanyan Xu, Minghang Wang, Yimin Guo, Xue Li, Lei Liu, Hongmei Yao, Hong Wen, Jianyou Chen, Xuemei Zhang, Zhiping Zhang, Li Yu, Dandan Chen, Wei Du, Qiaoli Chen, Hu Li, Wen Peng, Liting Zhang, Jiaman Tang, Baojuan Liu, Chen Zhan, Lianrong Huang, Xiaomei Chen

**Affiliations:** 1 Guangzhou Institute of Respiratory Health, State Key Laboratory of Respiratory Disease, National Clinical Research Center for Respiratory Disease, National Center for Respiratory Medicine, The First Affiliated Hospital of Guangzhou Medical University, Guangzhou Medical University, 151 Yanjiang Rd., Guangzhou, 510120 China; 2 Department of Pulmonary and Critical Care Medicine, Huizhou Third People’s Hospital, Guangzhou Medical University, Huizhou, China; 3 The Second Affiliated Hospital of Guangzhou Medical University, Guangzhou, China; 4 Dongguan Third People’s Hospital, Dongguan, China; 5 The Second Hospital Zhejiang University School of Medicine, Hangzhou, China; 6 The Second Clinical College of Guangzhou University of Chinese Medicine, Guangdong Provincial Hospital of Chinese Medicine, Guangdong Provincial Academy of Chinese Medical Sciences, Guangzhou, China; 7 The First People’s Hospital of Yunnan Province, Kunming, China; 8 The First Affiliated Hospital of Henan University of Chinese Medicine, Zhengzhou, China; 9 Xijing Hospital, Fourth Military Medical University, Xi’an, China; 10 The Second Hospital of Hebei Medical University, Shijiazhuang, China; 11 West China School of Medicine/West China Hospital of Sichuan University, Chengdu, China; 12 Department of Pulmonary and Critical Care Medicine, Guizhou Provincial People’s Hospital, Guiyang, China; 13 Dongguan People’s Hospital, Dongguan, China; 14 Qingdao Municipal Hospital, Qingdao, China; 15 Xinqiao Hospital, Third Military Medical University (Army Medical University), Chongqing, China; 16 The People’s Hospital of Jiangmen, Jiangmen, China; 17 Department of Pulmonary and Critical Care Medicine, Tongji Hospital, School of Medicine, Tongji University, Shanghai, China; 18 The Second People’s Hospital of Shenzhen, Shenzhen, China; 19 Shenzhen People’s Hospital, Shenzhen, China; 20 Ruijin Hospital Affiliated to Shanghai Jiao Tong University School of Medicine, Shanghai, China; 21 National Heart and Lung Institute, Imperial College London, & Royal Brompton and Harefield Foundation NHS Trust, London, UK

**Keywords:** Asthma, Cough predominant asthma, Cough, Cough sensitivity, Airway inflammation

## Abstract

**Background:**

Asthma is a heterogeneous disease with variable symptoms, which presents with cough either as the sole or predominant symptom with or without wheezing. We compared the clinical and pathophysiological characteristics of cough predominant asthma (CPA), cough variant asthma (CVA) and classic asthma (CA) in order to determine any differential phenotypic traits.

**Methods:**

In 20 clinics across China, a total of 2088 patients were finally recruited, including 327 CVA, 1041 CPA and 720 CA patients. We recorded cough and wheezing visual analogue scale, Leicester cough questionnaire (LCQ) and asthma control test scores. Fractional exhaled nitric oxide (FeNO), induced sputum cell counts, and capsaicin cough challenge were also measured and compared.

**Results:**

CPA patients more frequently presented with cough as the initial symptom, and laryngeal symptoms (p < 0.001), had less symptoms related with rhinitis/sinusitis and gastroesophageal reflux (p < 0.05) than CA patients. Comorbidities including rhinitis and gastroesophageal reflux were similar, while the proportion of COPD and bronchiectasis was higher in CA patients. There were no differences in FeNO levels, sputum eosinophil and neutrophil counts, FEV1 (%pred) decreased from CVA to CPA to CA patients (p < 0.001). Cough sensitivity was higher in CVA and CPA compared to CA (p < 0.001), and was positively correlated with LCQ scores.

**Conclusions:**

CVA, CPA and CA can be distinguished by the presence of laryngeal symptoms, cough sensitivity and airflow obstruction. Asthma-associated chronic cough was not associated with airway inflammation or comorbidities in our cohort.

*Trial registration* The Chinese Clinical Trial Registration Center, ChiCTR-POC-17011646, 13 June 2017

**Supplementary Information:**

The online version contains supplementary material available at 10.1186/s12931-022-02104-8.

## Introduction

Asthma is a chronic inflammatory disease of the airways. According to onset, triggers, clinical features, airway inflammation, response to treatment, and prognosis, asthma can be divided into different phenotypes, such as early onset asthma, severe asthma, classic asthma or atypical asthma [1, 2]. Asthma is classically characterized by variable episodes of shortness of breath, chest tightness, wheezing and cough. Although breathlessness and wheeze are the more frequent symptoms of asthma, cough can also be most troublesome major complaint [3]. Cough variant asthma (CVA), first described by Corrao and colleagues, presents with cough as a sole presenting symptom associated with normal lung function [4]. Since then, CVA has been recognized as being a common cause of chronic cough [5, 6]. Another less-well recognized type of cough associated with asthma is cough presenting not as the sole symptom but as the predominant persistent symptom of asthma, associated with mild wheezing and/or dyspnea. This type of asthma has been referred to as cough predominant asthma (CPA) in order to distinguish them from CVA [7–9]. Similarly, CPA is also recognized as a common cause of chronic cough [9]. In CPA, cough can be persistent even after regular anti-inflammatory treatments are administered and could be an indicator of exacerbation and poor control of asthma [10–16]. Finally, in contrast to CPA, classical asthma (CA) presents itself predominantly with wheezing and/or dyspnea, with mild cough or no cough symptom.

Asthma is often accompanied by comorbidities such as allergic rhinitis (AR), chronic rhinosinusitis, and gastroesophageal reflux, which are also common causes of chronic cough [5, 17–20]. However, it is not certain whether these comorbidities are related to cough in asthmatic patients. CVA has been reported to have a similar eosinophilic inflammation and bronchial hyperresponsiveness but higher cough sensitivity compared to CA [21]. For CPA, cough is the predominant symptom, but it was often ignored in current questionnaires of asthma control such as Asthma Control Test (ACT) and Asthma Control Questionnaire (ACQ) [22, 23]. Furthermore, the differences in clinical features, airway inflammation and cough sensitivity between CVA, CPA and CA have not been studied because there has been no study that has compared these different asthma phenotypes within a single cohort.

In order to fill this knowledge gap, we conducted a prospective, multicenter, CPA Cohort (CPAC) study in 2016 in China in order to elucidate the characteristics of these 3 phenotypes of asthma defined by the presence or absence of cough as a symptom. Within this framework, our objectives were to describe the baseline profiles of CPA versus CVA and CA patients in terms of demographics, symptoms (respiratory and other), co-morbidities, airway inflammation, cough sensitivity and lung function.

## Materials and methods

### Study design

**Fig. 1:**
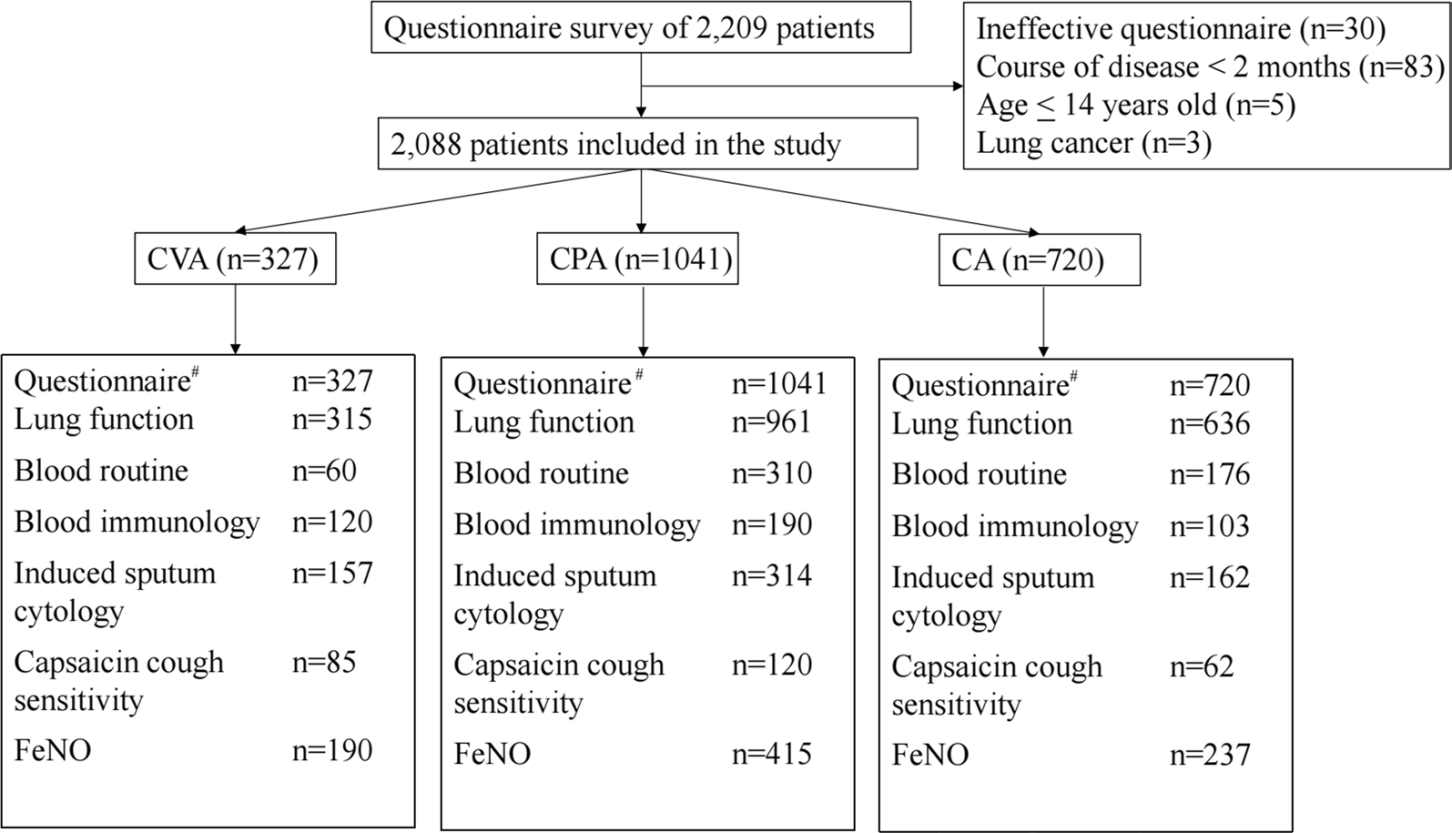
Flow chart of the study. ^#^Including demographics, respiratory symptoms, concomitant symptoms, comorbidities, smoking history and medications. CVA: cough variant asthma; CPA: cough predominant asthma; CA: classic asthma FeNO: Fractional exhaled nitric oxide

This was a prospective, multicenter, observational study that was conducted between February 2016 and March 2019, in 20 hospital centers from 11 provinces and municipalities across China (Additional file 1). The study flow chart is presented in Fig. 1. For all patients enrolled into this study, detailed medical history and physical examination were recorded in a standard case report file (Additional file 2), including demographics, respiratory symptoms, concomitant symptoms, comorbidities, smoking history and medications. Relevant questionaires including the asthma control test (ACT), cough symptom score (CSS), cough visual analogue scale (VAS) and Leicester Cough Questionnaire (LCQ) were completed. A total of 633 patients in 10 centers completed the induced sputum test, 842 patients from 16 centers had FeNO measurement and 267 patients from 3 centers underwent capsaicin cough challenge (details in Additional file 1).

This study was approved by the Ethics Committee of The First Affiliated Hospital of Guangzhou Medical University and the institutional review boards of each participating center (201604). It has also been registered in the Chinese Clinical Trial Registration Center (ChiCTR-POC-17011646). All participating patients provided written informed consent.

### Subjects

Patients aged 14 years old or older were recruited amongst those attending the recruiting centres with a history of wheeze or dyspnea and/or cough and those with a history of chronic cough alone. All patients were diagnosed as asthma by physicians according to the Global Strategy for Asthma Management and Prevention Guidelines (GINA Guidelines, 2016), and the Chinese Guidelines of Diagnosis and Management of Chronic Cough [24, 25]. On the basis of the presence of bronchial hyperresponsiveness [fall in forced expiratory volume in 1 s (FEV1) from baseline of ≥ 20% with increasing doses of inhaled methacholine], or of a positive bronchodilator test (increase in FEV1 ≥ 12% and ≥ 200 mL from baseline), CVA was diagnosed if the patient presented with cough as the sole or main symptom lasting more than 8 weeks without wheeze and dyspnea; CPA was diagnosed if the patient presented with cough as the predominant symptom lasting for more than 8 weeks in addition to transient wheezing and/or dyspnea, and CA was diagnosed if the patient had wheezing and/or dyspnea as the main symptom(s), with or without cough (Table 1 and Additional file 3). Co-morbidities were determined according to history and on previous diagnosis made by other physicians. Patients with an acute asthma attack or an acute upper respiratory tract infection within 8 weeks of recruitment were excluded, as well as those with serious systemic diseases, pregnancy, and breast-feeding.

**Table 1:** The definition of CVA, CPA and CA

Diseases	Definition	
	Clinical symptoms	Laboratory tests
CVA	Cough as the sole or main symptom lasting more than 8 weeks without wheeze and dyspnea	Normal ventilation function positive bronchodilator test^※^ or positive bronchial challenge test^&^
CPA	Cough as the predominant symptom lasting for more than 8 weeks and transient wheezing and/or dyspnea	Positive bronchodilator test^※^ or positive bronchial challenge test^&^
CA	Wheezing and/or dyspnea as the main symptom(s), with or without cough	Positive bronchodilator test^※^ or positive bronchial challenge test^&^

*CVA* Cough variant asthma, *CPA* Cough predominant asthma, *CA* Classic asthma

^※^Increase in FEV1 of > 12% and > 200 mL from baseline

^&^Fall in FEV1 from baseline of > 20% with standard doses of methacholine or histamine

### Assessment

The details of cough and wheezing VAS scores, cough symptom score (CSS) and Leicester Cough Questionnaire (LCQ) are provided in Additional file 2. In brief, laryngeal sensitivity was assessed by presence of laryngeal symptoms and the ACT was used for evaluating asthma control [22]. Separate cough and wheezing VAS scores were obtained on 100 mm scales on which patients indicated the severity of cough or wheezing, with 0 indicating no cough or wheezing, and 100 indicating the worst cough or wheezing. The cough symptom score (CSS) consists of two questions about the subjective recognition of cough frequency and severity duting the day and night. The scores for each question range from 0 to 5 [26, 27]. Cough-related quality of life was assessed by the Leicester Cough Questionnaire (LCQ), which contains19 items divided into three domains (physical, psychological and social) [28].

## Methods

Spirometry and bronchial challenge were performed according to the current ATS/ERS guidelines [29, 30]. The provocative cumulative dose of methacholine causing a 20% fall in FEV1 (PD20-FEV1) less than 2.500 mg was used as a marker for bronchial hyperresponsiveness (BHR) [30]. Sputum was induced and processed as described by the Chinese Guidelines for Diagnosis and Management of Cough (2015) [25]. Briefly, sputum was induced with 3% saline, and sputum was mixed with four times its volume of 0.1% dithiothreitol. The cell smear was stained with hematoxylin–eosin. Differential cell counts were obtained by counting 400 non-squamous cells. FeNO measurements were performed in accordance with the standard procedure as previously described [31]. Cough sensitivity was measured by a single breath inhalation capsaicin test with a compressed air-driven nebulizer controlled by a breath-activated dosimeter. Briefly, doubling concentrations of capsaicin solutions (1.95–1000 μmol/L) were inhaled at 1 min intervals, and coughs were counted in the first 30 s after inhalation. The lowest concentrations of capsaicin (C2) which evoked two coughs were obtained and the level of cough reflex sensitivity (CRS) was presented as the logarithm of C2 (logC2). The lowest concentrations of capsaicin (C5) which evoked five or more coughs were obtained and the level of CRS was presented as the logarithm of C5 (logC5) [25].

### Statistical analysis

SPSS 23.0 software was used for analysis. Age, FEV1% pred, body mass index (BMI), cough day and night integral values were expressed as means ± standard deviation. Due to non-normal distributions, the duration of disease, blood eosinophil counts, FeNO values, induced sputum cytology, logC5, cough VAS score, LCQ scores and ACT scores were presented as medians (interquartile range). When the measurement data for two or more groups presented with normal distributions, two independent sample t-tests or one-way analysis of variance (ANOVA) was used. Otherwise, Mann–Whitney U tests or multi-sample Kruskal–Wallis H test for independent samples were performed. The comparison of categorical variables was performed by the χ^2^ test or Fisher’s exact probability method. We used the Bonferroni correction method for the comparisons in two-group analyses. Two-sided p < 0.05 indicated statistical significance.

## Results

### Baseline characteristics

A total of 2088 patients with a mean age of 45.4 ± 14.5 years, were enrolled in the study, and were divided into 1041 patients with CPA, 720 with CA and 327 with CVA (Fig. 1 and Table 2). The CVA patients were younger than the CPA and CA patients (all p < 0.05). There were more females in the CVA and CPA patients than that in the CA patients (all p < 0.05). The median duration of disease increased from CVA to CPA to CA patients (p < 0.001), so did for the proportion of smoking (p < 0.001). A total of 549 (26.3%) patients, including 34 CVA, 274 CPA and 241 CA, were treated with inhaled corticosteroids (ICS)/ICS + long-acting Beta2-agonists (LABA) regularly before enrollment in the past 3 months. A total of 1425 (68.2%) patients were not prescribed asthmatic medication, and 114 (5.5%) patients could not report their treatment status (Additional file 4). More CA patients received regular antiasthmatic treatment compared to CPA and CVA patients (p < 0.001). The proportion of patients with dry cough in the CVA patients was higher than that in CPA patients (p < 0.001). As compared with CA patients, more CPA patients presented with cough as the initial symptom (77.1% vs 31.4%, p < 0.001) with a longer time from start of cough to first wheezing or dyspnea (p = 0.013) (Table 2).

**Table 2:** Clinical characteristics of CVA, CPA and CA

Variable	Total	CVA	CPA	CA	p
Number	2088	327	1041	720	–
Age, years	45.4 ± 14.5	43.5 ± 15.7*^#^	45.8 ± 14.3	46.0 ± 14.0	0.016
Female, n (%)	1254 (60.1)	204 (62.4)^#^	644 (61.9)^#^	406 (56.4)	0.045
BMI kg/m^2^	23.3 ± 3.6	22.8 ± 3.6	23.4 ± 3.7	23.6 ± 3.5^&^	0.017
Asthmatic duration, month	24.5 (7.0, 96.0)	12.0 (3.5, 36.0)*^#^	24.0 (6.0, 84.0)^&#^	48.0 (12.0, 120.0)^&^*	< 0.001
Non-smoker, n (%)	1632 (78.2)	289 (88.4)*^#^	812 (78.0)	531 (73.8)	< 0.001
Ex-smoker, n (%)	101 (4.8)	4 (1.2)*^#^	56 (5.4)	41 (5.7)	< 0.001
Current smoker, n (%)	355 (17)	34 (10.4)^#^	173 (16.6)^#^	148 (20.6)	< 0.001
Blood Eos (10^9^/L)	0.2 (0.1, 0.5)	0.3 (0.1, 0.4)	0.3 (0.1, 0.5)	0.2 (0.1, 0.4)	0.312
With regular treatment^†^ within the past 3 months, n (%)	549 (26.3)	34 (10.4)*^#^	274 (26.3)^&#^	241 (33.5)^&^*	< 0.001
Number of patients with cough, n (%)	1851 (88.6)	327 (100)	1041 (100)	483 (67.1)	NS
Dry cough, n (%)	1045/1851 (56.5)	222/327 (67.9)*	544/1041 (52.3)	279/483 (57.8)	< 0.001
Day cough, n (%)	1079/1851 (58.3)	235/327 (71.9)*^#^	579/1041 (55.6)	266/483 (55	< 0.001
Night cough, n (%)	1505/1851 (81.3)	258/327 (78.9)	869/1041 (83.5)	378/483 (78.3)	0.312
Cough as the initial symptom, (n) %	1356 (64.9)	327 (100)	803 (77.1)	226 (31.4)*	< 0.001
Time from cough to wheeze ^^^, month	–	–	3.0 (1.0,12.0)^#^	2.0 (1.0, 8.0)	0.032
Lung function, n	1913	314	961	638	NS
FEV1% pred	78.8 ± 20.7	92.6 ± 12.8*^#^	77.8 ± 20.8^&#^	73.7 ± 20.8^&^*	< 0.001
FVC% pred	92.9 ± 17.6	96.5 ± 14.9*^#^	92.6 ± 18.5	91.7 ± 17.2	< 0.001
FEV1/FVC%	70.8 ± 13.2	80.7 ± 7.9*^#^	70.0 ± 12.6^&#^	67.0 ± 13.7^&^*	< 0.001
MMEF% pred	48.5 ± 24.9	66.4 ± 21.6*^#^	46.4 ± 23.8^&#^	43.0 ± 24.0^&^*	< 0.001

Data were presented as percentage or mean SD or median (IQR)

CVA: Cough variant asthma; CPA: Cough predominant asthma; CA: Classic asthma; BMI: body mass index; FEV1% pred: forced expiratory volume in 1 s in % predicted; FVC% pred: forced vital capacity in % predicted; MMEF% pred: the maximum mid-expiratory flow in % predicted

^†^Inhaled corticosteroids (ICS)/ICS + long-acting Beta2-agonists (LABA)

^^^How long the patient developed wheezing after developing cough as the initial symptom of asthma onset in CPA and CA patients

^&^Compared to CVA, p < 0.05

*Compared to CPA, p < 0.05

^#^Compared to CA, p < 0.05

### Asthma control and cough score

As shown in Table 3, the ACT score in the CPA patients was similar to that in CA patients and both of them were lower when compared to the CVA patients (p < 0.001). CPA patients showed higher cough VAS compared to CA patients (p < 0.001). The daytime CSS of CPA and CVA patients was higher than that of CA patients (p < 0.001). Furthermore, CPA patients had lower LCQ scores than CA patients. There was no significant difference in cough VAS scores between CPA and CVA patients (p = 0.062) (Table 3).

**Table 3:** ACT and cough scores in CVA, CPA and CA

	CVA	CPA	CA	p
ACT score	21 (18, 22.3)*^#^	18 (15, 21)	18 (15, 21)	< 0.001
Cough VAS	50 (30, 60)	50 (30, 70)	10 (0, 30)^&^*	< 0.001
Daytime CSS	2 (2, 3)	2 (2, 3)	1 (0, 2)^&^*	< 0.001
Nighttime CSS	1 (1, 2)*^#^	2 (1, 3)^&#^	0 (0, 1)^&^*	< 0.001
LCQ	14.3 (11.7, 16.9)*^#^	13.6 (11.0, 16.6)^&#^	18.7 (14.8, 21.0)^&^*	< 0.001
LCQ-physiological	4.8 (4.0, 5.5)	4.5 (3.8, 5.3)	6.0 (4.6, 7.0)	< 0.001
LCQ-psychological	4.6 (3.6, 5.5)	4.4 (3.4, 5.6)	6.4 (14.7, 7.0)	< 0.001
LCQ-social	5 (3.8, 6.3)	5 (3.8, 6.0)	6.8 (15.0, 7.0)	< 0.001

Data were presented as median (IQR)

CVA: cough variant asthma; CPA: cough predominant asthma; CA: classic asthma; ACT: asthma control test; VAS: visual analogue scale; CSS: cough symptom score; LCQ: Leicester cough questionnaire

^&^Compared to CVA, p < 0.05

*Compared to CPA, p < 0.05

^#^Compared to CA, p < 0.05

### Accompanying symptoms and comorbidities

The proportion of patients with laryngeal symptoms in CVA and CPA patients were significantly higher than that in CA patients (all p < 0.001). A lower proportion of patients with nasal symptoms and reflux symptoms was observed in the CVA patients as compared with that in CPA and CA patients (all p < 0.05) (Table 4). CA patients had a higher prevalence of comorbidities, including chronic obstructive pulmonary disease (COPD) and bronchiectasis, followed by CPA (p < 0.05). There were more patients with sinusitis among the CA and CPA patients than in the CVA patients (all p < 0.05) (Table 5).

**Table 4:** Nasal, laryngeal and oesophageal symptoms in CVA, CPA and CA

Accompanying symptoms (%)	CVA	CPA	CA	p
Numbers	320	1011	443	–
Any one of laryngeal symptoms, n (%)	244 (76.3)*^#^	764 (75.6)^&#^	291 (65.7)	< 0.001
Itchy throat, n (%)	189 (59.1)^#^	571 (56.5)^#^	195 (44.0)	< 0.001
Itching below the pharynx, n (%)	10 (3.1)*^#^	122 (12.1)	49 (11.1)	< 0.001
Sore throat, n (%)	28 (8.8)*^#^	208 (20.6)	81 (18.3)	< 0.001
Abnormal sensation of throat, n (%)	77 (24.1)	300 (29.7)	133 (30.0)	0.122
Requent throat clearing, n (%)	105 (32.8)	329 (32.5)	119 (26.9)	0.077
Any one of nasal symptoms, n (%)	179 (55.9)*^#^	654 (64.7)	280 (63.2)	0.018
Mucus adherence post laryngeal wall, n (%)	79 (24.7)	318 (31.5)	134 (30.2)	0.069
Stuffy nose, n (%)	68 (21.3) *^#^	332 (32.8)	139 (31.4)	< 0.001
Itchy nose, n (%)	73 (22.8) *^#^	290 (28.7)	136 (30.7)	0.048
Sneeze, n (%)	81 (25.3)	218 (21.6)^#^	124 (28.0)	0.024
Runny nose, n (%)	54 (16.9)	329 (32.5)	152 (34.3)	< 0.001
Postnasal drip, n (%)	30 (9.4)	86 (8.5)	39 (8.8)	0.890
Any reflux symptoms, n (%)	73 (22.8) *^#^	307 (30.4)	148 (33.4)	0.006
Acid reflux, n (%)	35 (10.9)	142 (14.0)	65 (14.7)	0.283
Belching, n (%)	24 (7.5)	104 (10.3)	42 (9.5)	0.335
Nausea, n (%)	28 (8.8)	135 (13.4)	55 (12.4)	0.091
Upset stomach, n (%)	28 (8.8)*^#^	141 (13.9)^&#^	84 (19.0)^&^*	< 0.001
Heartburn, n (%)	9 (2.8)*^#^	90 (8.9)	40 (9.0)	0.001

CVA: cough variant asthma; CPA: cough predominant asthma; CA: classic asthma

^&^Compared to CVA, p < 0.05

*Compared to CPA, p < 0.05

^#^Compared to CA, p < 0.05

**Table 5:** Comorbidities in CVA, CPA and CA

Comorbidities	CVA	CPA	CA	*p*
Number	296	945	578	–
Rhinitis, n (%)	127 (42.9)	422 (44.7)	255 (44.1)	0.868
Sinusitis, n (%)	51 (17.2)*^#^	253 (26.8)	141 (24.4)	0.004
COPD, n (%)	0 (0.0)*^#^	70 (7.4)^&#^	64 (11.1)^&^*	< 0.001
Bronchiectasis, n (%)	5 (1.7)^#^	28 (3.0)^#^	33 (5.7)	0.003
Gastroesophageal reflux, n (%)	27 (9.1)	115 (12.2)	49 (8.5)	0.052

COPD: chronic obstructive pulmonary disease; CVA: cough variant asthma; CPA: cough predominant asthma; CA: classic asthma

^&^Compared to CVA, p < 0.05

*Compared to CPA, p < 0.05

^#^Compared to CA, p < 0.05

### Lung function and airway inflammation

**Fig. 2:**
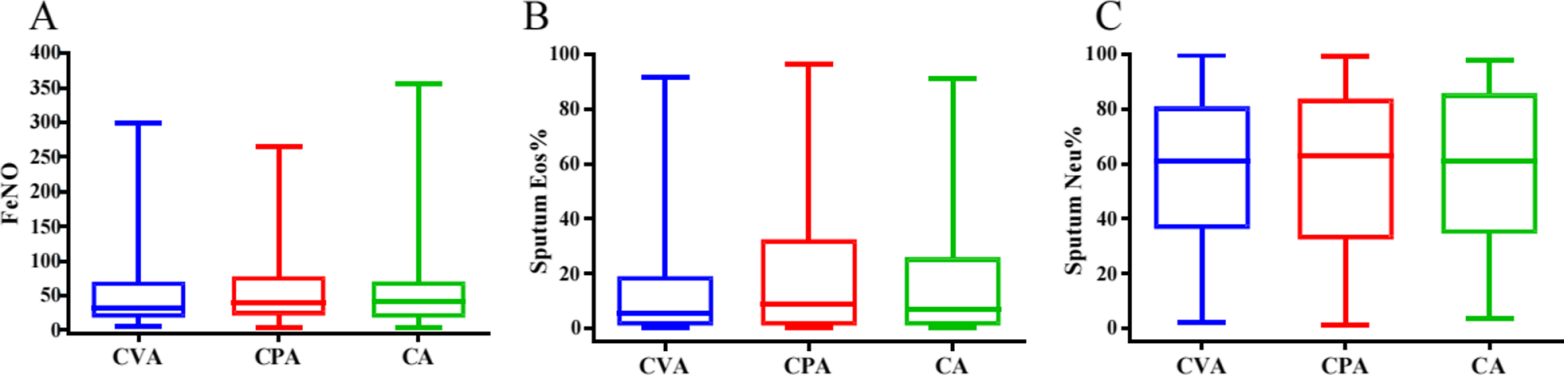
Airway inflammation in CVA, CPA and CA. **A** Fractional exhaled nitric oxide (FeNO) in CVA, CPA and CA; **B** Percentage of eosinophils in sputum in CVA, CPA and CA; **C** Percentage of neutrophils in sputum in CVA, CPA and CA. CVA: cough variant asthma; CPA: cough predominant asthma; CA: classic asthma

FEV1, FEV1/FVC (%) and the maximum mid-expiratory flow in % predicted (MMEF% pred) were lowest in CA group, followed by CPA and CVA (all p < 0.05). Forced vital capacity in % predicted (FVC % pred) of CVA patients was significantly higher than that of CPA and CA patients (all p < 0.05) (Table 2). No significant differences were found in eosinophil (Eos %) and neutrophil (Neu %) counts in sputum and FeNO levels between the three groups (Fig. 2).

### Cough sensitivity

**Fig. 3:**
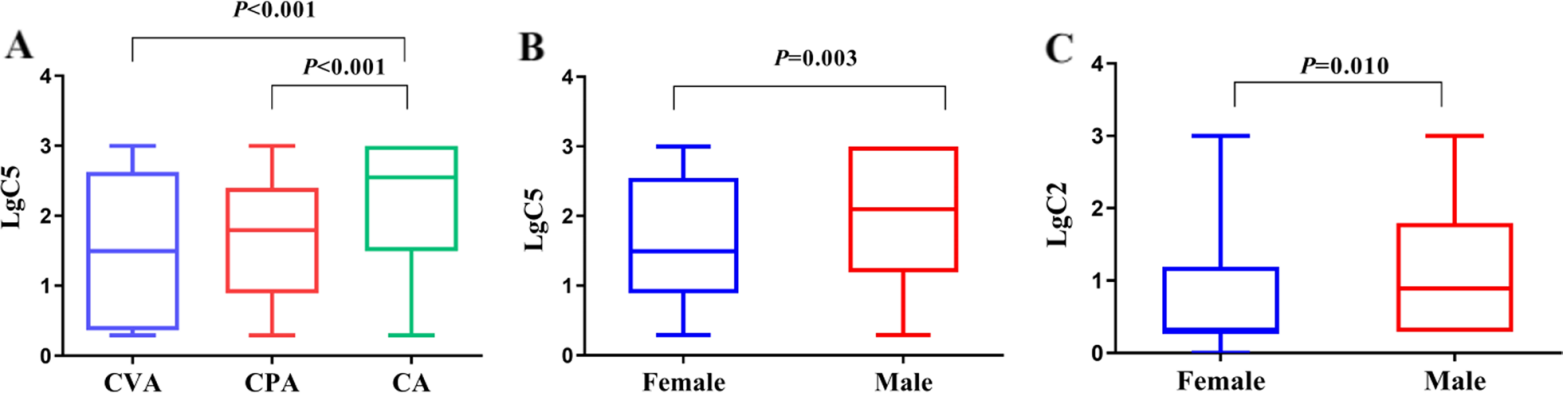
Capsaicin cough sensitivity. **A** logC5 in CVA, CPA and CA; **B** logC5 in females and males for total patients (n = 534); **C** logC2 in females and males for total patients (n = 534). CVA: cough variant asthma; CPA: cough predominant asthma; CA: classic asthma. logC2: the lowest concentrations of capsaicin (C2) which evoked two coughs were obtained and the level of cough reflex sensitivity (CRS) was presented as the logarithm of C2 (logC2); logC5: The lowest concentrations of capsaicin (C5) which evoked five or more coughs were obtained and the level of CRS was presented as the logarithm of C5 (logC5)

A total of 264 patients completed the capsaicin cough challenge test, including 84 CVA, 120 CPA and 60 CA patients. CVA and CPA patients showed similar values of logC5, but lower values than that of the CA patients (1.5, 1.8 vs. 2.5 all p < 0.001) (Fig. 3). For all patients, the cough sensitivity of females was higher than that of males (p < 0.05) (Fig. 3). LogC5 of capsaicin cough sensitivity was positively correlated with LCQ scores (r = 0.416, p < 0.001; n = 183), but eosinophil (r = 0.002, p = 0.510; n = 230) and neutrophil (r = 0.015, p = 0.060; n = 230) counts in induced sputum were not correlated with logC5.

## Discussion

Our study, for the first time, divided asthma into the three phenotypes of CVA, CPA and CA based on the presence and severity of cough in relationship to the presence of other asthmatic symptoms of wheeze and chest tightness and compared the clinical and pathophysiological characteristics. We found that CPA patients had a higher proportion of females, usually presenting initially with cough predominantly. There were no significant differences with regard to comorbidities related with cough, reflux-related symptoms, rhinitis/sinusitis-related symptoms, and sputum eosinophil and neutrophil counts and FeNO levels between CPA and CA. However, the proportion of those with laryngeal symptoms was higher in CPA compared to CA. Cough sensitivity in CPA patients was similar to that in CVA patients, but significantly higher than that in CA patients. In addition, from the asthma severity point of view, both CPA and CA were less well-controlled and had evidence of airflow obstruction compared to CVA. Thus, our study confirms that CVA, CPA and CA phenotypes are distinct in terms of the features of asthma, airflow obstruction and cough severity as well as cough sensitivity.

CPA had a similar female predominance as CVA, as we previously reported [32]. In a worldwide survey of patients attending cough clinics, chronic cough patients were usually found to be middle-aged females who had heightened cough sensitivity compared to males [33].

Cough is one of the most common symptoms reported by asthmatic patients. Although CPA patients showed lower wheezing scores and better lung function, they had higher cough scores and lower LCQ scores than CA, indicating worse quality of life due to cough. Furthermore, there was no significant difference in ACT scores between CPA and CA, indicating that chronic cough caused an impairment of quality of life while ACT scores were not different. This may not be surprising because the ACT does not specifically request information on cough as a symptom of asthma, despite the recognition that cough can be a troublesome as is breathlessness in asthmatic patients [3]. Cough can also be an indicator for exacerbation and poor control [15]. Furthmore, we found that cough remained a prominent symptom in spite of improvement of wheezing in those patients who experienced regular anti-asthma treatment in last 3 months. Therefore, more attention should be paid to cough in the management of asthma.

The proportion of current smokers, COPD and bronchiectasis in CPA was significantly lower than that in CA patients, indicating that smoking, COPD and bronchiectasis could not fully explain the chronic cough of CPA. Rhinitis/sinusitis and gastroesophageal reflux are common causes of chronic cough [5, 34]. Our results showed that CPA patients had similar proportions of sinusitis, rhinitis and gastroesophageal reflux as CA patients. Therefore, the chronic cough in CPA would seem unlikely to be associated with sinusitis, rhinitis and gastroesophageal reflux. In addition, there were no significant differences with regard to the proportion of rhinitis and gastroesophageal reflux between CVA and CA patients, and a lower prevalence of sinusitis and bronchiectasis in CVA patients. It is also worth noting that abnormal laryngeal sensations such as itchy throat, throat clearing and irritation and triggers of cough are common in patients with chronic cough [35, 36], supporting the presence of cough hypersensitivity [36, 37]. Also, the proportion of patients with laryngeal symptoms in CVA and CPA was higher than that in CA patients, which is in line with the higher capsaicin cough sensitivity we report in CVA and CPA patients. Based on the above results, the chronic cough of CPA is underlined by the presence of cough hypersensitivity.

Chronic cough associated with asthma and non-asthmatic eosinophilic bronchitis typically respond well to therapy with corticosteroids, thus leading to the general assumption that the suppression of eosinophilic airway inflammation is the cause of basis for the improvement in cough [38, 39]. However, recent studies have challenged the causal relationship between eosinophilic airway inflammation and cough in asthmatics [40, 41], such as the failure of anti-interleukin (IL)-5 antibody to modify the cough of severe asthma [41], and the possibility for a role of activated mast cells in cough rather than eosinophilic airway inflammation [42, 43]. Similarly, in our cohort, there were no significant differences in the sputum eosinophil and neutrophil counts and FeNO levels among CVA, CPA and CA, which indicates that the measured severity of airway inflammation was not related to cough in asthma. This observation would also indicate that the severity of cough should be used as a measure of asthma control, independent of eosinophilic inflammation.

We found that the cough sensitivity of CPA and CVA was significantly higher than that of CA, supporting a role for the increased cough sensitivity in the pathogenesis of cough in asthma. The mechanism of increased cough sensitivity in asthmatic patients is currently unclear. Cough sensitivity in patients with asthma and COPD has been related to the severity of cough, but not to the degree of airflow obstruction [44]. Cough sensitivity of asthmatic patients could be decreased after inhaled corticosteroids [45]. It was possible that this effect was specific to the provocation agent utilized. In that study, mannitol, targeting on mast cell induced cough, was utilized as the provocative agent [45]. which would be inhibited by steroids. However, cough sensitivity of patients with asthma and COPD sometimes may not decrease after inhaled corticosteroids [44, 46, 47], but responded to anticholinergic treatment [44]. Testing cough sensitivity by capsaicin or citric acid is a direct measure of cough neural sensitivity. Previous studies have shown that patients with CVA had increased cough sensitivity to capsaicin [44, 48, 49]. Moreover, female patients and patients aged > 50 years had higher cough sensitivity to capsaicin than male patients and patients aged < 50 years, respectively [32]. The increased cough sensitivity caused by OVA-sensitized airway inflammation may be related to the expression of the transient receptor potential vanilloid 1 (TRPV1) in lung sensory nerve cells [50]. The overexpression of functional TRPV1 channels in the airway epithelium of patients with refractory asthma may provide a new therapeutic target for such asthma [51]. The mechanism underlying the increased cough sensitivity in CPA needs further study. However, P2X3 antagonist may be beneficial in the cough hypersensitivity of asthma [52].

The current questionnaires used to assess asthma control pay little attention to cough symptoms and their impact on quality of life. ACT and asthma control questionnaire (ACQ) scores are important indicators used to evaluate asthma control levels in the GINA guidelines [24], but they do not include questions relating to cough severity. In the ACT score of the asthma control test, one of the five questions relates to asthma symptoms which are either not defined nor mention cough as one of the symptoms [22]. In the other asthma control questionnaire (ACQ), cough is not asked about separately [23]. Some asthmatic patients have a higher cough frequency but a lower ACQ score [12], which may have resulted because of the lack of emphasis on assessing the severity of cough. The LCQ is widely used to assess the impact of cough on quality of life [53]. In patients with severe asthma, the LCQ score was moderately correlated with the ACQ-6 and asthma quality of life questionnaire (AQLQ) scores [54]. In the current study, we combined the ACT, cough VAS and LCQ scores to obtain a more comprehensive evaluation of asthma control that includes the impact of cough on quality of life.

Our study has some limitations. First, medical history and symptom assessment were assessed by questionnaires, which may lead to recall bias. Second, induced sputum tests, FeNO measurements and cough challenges were not conducted in all enrolled patients, this cohort nevertheless represents the largest sample of CVA and CPA patients studied. Third, a small number of patients were found to have concomitant COPD or bronchiectasis, that could have contributed to chronic cough symptoms. Finally, the details of drug name and dose of regular anti-asthma treatment were not collected in the questionnaires. However, we took the effect of treatment into consideration, which was important because inhaled corticosteroids could be effective in controlling asthma symptoms including cough. We divided the patients into groups according to whether they received regular treatment or not in the past 3 months, and found that though there was lower VAS scores in CPA patients with regular treatment, cough still was a prominent symptom, and there was no significant difference in cough VAS scores between those who received regular treatment and those who did not receive regular treatment in CVA, CA patients, indicating that the difference of the proportion of patients who received anti-asthma treatments between CA, CPA/CVA could not explain the presence of cough.

## Conclusions

Our analysis supports the concept that CVA, CPA and CA represent distinct phenotypes of asthma when defined according to the presence of cough as a predominant symptom in association with wheeze and/or dyspnea. Asthmatic cough is more likely to be related to cough hypersensitivity rather than to comorbidities and airway inflammation. Our study also highlights the need to include an assessment of the severity of cough which could also be a marker of the severity of asthma independent of eosinophilic inflammation.

## Supplementary Information

**Additional file 1.** List of all clinical centers participating in the study.

**Additional file 2.** Case Report Form of the study.

**Additional file 3.** Questions for indicating diagnosis of CVA, CPA and CA from questionnaire.

**Additional file 4.** Comparison of clinical characteristics among CVA, CPA and CA patients with regular and without regular treatment.

## Data Availability

The datasets used and/or analyzed during the current study are available from the corresponding author on reasonable request.

## Abbreviations

CVA: Cough variant asthma
CPA: Cough predominant asthma
CA: Classic asthma
VAS: Visual analogue scale
LCQ: Leicester cough questionnaire
ACT: Asthma control test
FeNO: Fractional exhaled nitric oxide
FEV1% pred: The forced vital capacity in percent predicted values
AR: Allergic rhinitis
CRS: Cough reflex sensitivity
CPAC: CPA Cohort
CSS: Cough symptom score
GINA: Asthma Management and Prevention Guidelines
BHR: Bronchial hyperresponsiveness
ICS: Inhaled corticosteroids
LABA: Long-acting Beta2-agonists
C5: The lowest concentrations of capsaicin which evoked five or more coughs
LogC5: Logarithm of C5
BMI: Body mass index
ANOVA: One-way analysis of variance
COPD: Chronic obstructive pulmonary disease
FVC % pred: The forced vital capacity in percent predicted values
MMEF % pred: The maximum mid-expiratory flow in percent predicted values
Eos (%): Percentage of eosinophils
Neu (%): The percentage of neutrophils
ACQ: Asthma control questionnaire
AQLQ: Asthma quality of life questionnaire
TRPV1: The transient receptor potential vanilloid 1
