# Supplementary material for: Characteristics of different asthma phenotypes associated with cough: a prospective, multicenter survey in China
Source: Respir Res. 2022 Sep 12;23:243. doi: 10.1186/s12931-022-02104-8 (PMC9469623; doi:10.1186/s12931-022-02104-8)
Supplement: Supplementary file 1 — Additional file 1. List of all clinical centers participating in the study. [file 12931_2022_2104_MOESM1_ESM.docx]

**Supplement 1**

**List of all clinical centers participating in the study**

| No. | Centers | N | Lung function | Induced sputum cytology | Capsaicin cough sensitivity | FeNO |
| --- | --- | --- | --- | --- | --- | --- |
| 1 | The First Affiliated Hospital of Guangzhou Medical University | 452 | 335 | 323 | 250 | 274 |
| 2 | Huizhou The Third People’s Hospital | 168 | 167 | 78 | 0 | 25 |
| 3 | The Second Affiliated Hospital of Guangzhou Medical University | 138 | 138 | 114 | 0 | 123 |
| 4 | Dongguan Third People's Hospital | 132 | 132 | 2 | 0 | 3 |
| 5 | The Second Hospital Zhejiang University School of Medicine | 124 | 98 | 0 | 0 | 82 |
| 6 | The Second Clinical College of Guangzhou University of Chinese Medicine | 114 | 113 | 1 | 0 | 0 |
| 7 | The First People's Hospital of Yunnan Province | 104 | 101 | 60 | 0 | 87 |
| 8 | The First Affiliated Hospital of Henan University of Chinese Medicine | 99 | 63 | 0 | 0 | 17 |
| 9 | Xijing Hospital, Fourth Military Medical University | 97 | 94 | 0 | 0 | 41 |
| 10 | The Second Hospital of Hebei Medical University | 86 | 86 | 0 | 0 | 1 |
| 11 | West China Hospital of Sichuan University | 85 | 82 | 35 | 5 | 80 |
| 12 | Guizhou Provincial People's hospital | 84 | 79 | 0 | 0 | 23 |
| 13 | Dongguan People's Hospital | 74 | 53 | 1 | 0 | 8 |
| 14 | Qingdao Municipal Hospital | 62 | 62 | 0 | 0 | 0 |
| 15 | Xinqiao Hospital, Third Military Medical University (Army Medical University) | 57 | 57 | 3 | 0 | 24 |
| 16 | The People's Hospital of Jiangmen | 52 | 52 | 0 | 0 | 0 |
| 17 | Tongji Hospital of Tongji University | 49 | 48 | 16 | 12 | 31 |
| 18 | The Second People's Hospital of Shenzhen | 42 | 39 | 0 | 0 | 0 |
| 19 | Shenzhen People's Hospital | 35 | 34 | 0 | 0 | 5 |
| 20 | Ruijin Hospital Affiliated to Shanghai Jiao Tong University School of Medicine | 34 | 34 | 0 | 0 | 18 |
|  | Total | 2088 | 1867 | 633 | 267 | 842 |
