# Supplementary material for: Characteristics of different asthma phenotypes associated with cough: a prospective, multicenter survey in China
Source: Respir Res. 2022 Sep 12;23:243. doi: 10.1186/s12931-022-02104-8 (PMC9469623; doi:10.1186/s12931-022-02104-8)
Supplement: Supplementary file 2 — Additional file 2. Case Report Form of the study. [file 12931_2022_2104_MOESM2_ESM.docx]

**Supplement 2**

**Case Report Form**

Medical history and symptom assessment at baseline

Date: yyyy/mm/dd

| Name |  | Gender |  | Date of birth |  | Nationality |  |
| --- | --- | --- | --- | --- | --- | --- | --- |
| Address |  | | | Contact |  | | |

1. Smoking history：

□Never smoke

□Smoking：Duration: ___ month; amount packs/day □ Quit smoking month

1. Duration of disease ___ month
2. Since the onset of illness, did you have the following symptoms (choose one or more)?

□ Wheeze □ Dyspnea □Cough □Chest tightness

1. Wheeze/dyspnea Visual Analogue Scale (VAS):


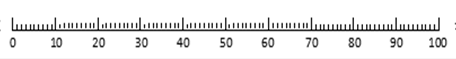


1. Since the onset of illness, what were the predominant symptoms (choose one or more)?

□Wheeze □ Dyspnea □Cough □Chest tightness

1. Since the onset of illness, what was the most predominant symptom that troubled you (choose one)?

□Wheeze □ Dyspnea □Cough □Chest tightness

1. In the initial stages of asthma, before wheezing or dyspnea, was cough the first symptom? □Yes □No
2. If you chose "Yes" in question 7, how long did it take for you to have wheezing or dyspnea after coughing? month
3. Did you experience cough symptoms before each wheezing or dyspnea or an acute attack?

□Yes □No

1. If you chose "Yes" in question 9, how long did it take for you to wheeze or have dyspnea after coughing? ___ day
2. Since the onset of illness, whether cough was the main symptom for a certain period of time (≥8 weeks)?

□Yes, there are now □Yes, in the past □No

1. Since the onset of illness, how long has the cough as the main symptom lasted for the longest time? ___ month
2. Duration of the time of cough: □0 week □≤3 weeks □3~8weeks □>8weeks
3. Cough VAS:


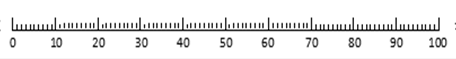


1. Current cough symptom score Day time: Night time: ___

Day Cough Symptom Score

0 = no cough during the day

1 = cough for one short period

2 = cough for more than two short periods

3 = frequent coughing, which did not interfere with usual daytime activities

4 = frequent coughing, which did interfere with usual daytime activities

5 = distressing coughs most of the day

Night cough symptom score

0 = no cough during the night

1 = cough on waking only

2 = wake once or early due to cough

3 = frequent waking due to coughs

4 = frequent coughs most of the night

5 = distressing coughs preventing any sleep

1. When does your cough often occur?

□Day time □Before going to bed □Night □Morning

1. Cough property (in the last 4 weeks):

□Dry cough □Productive cough (sputum ≥10 ml per day)

Sputum property □Mucoid □Foamy □Watery

Sputum color □White □Yellow □Yellow and white □ other

□Cough before wheezing □Cough during wheezing □Cough after wheezing relieved

□There was no obvious relationship between cough time and wheezing time

1. Current accompanying symptoms and severity of accompanying symptoms

| Accompanying symptom  □No □Yes | Mild | Moderate | Severe |
| --- | --- | --- | --- |
| □Itchy throat |  |  |  |
| □Itching below the pharynx |  |  |  |
| □Sore throat |  |  |  |
| □Foreign body sensation in pharynx |  |  |  |
| □Mucus attachment to the posterior pharyngeal wall |  |  |  |
| □Frequent throat clearing |  |  |  |
| □Stuffy nose |  |  |  |
| □Itchy nose |  |  |  |
| □Sneeze |  |  |  |
| □ Runny nose |  |  |  |
| □ Yellow nasal mucus |  |  |  |
| □ Postnasal drip |  |  |  |
| □Acid reflux |  |  |  |
| □Belching |  |  |  |
| □Nausea |  |  |  |
| □Upset stomach |  |  |  |
| □Burning behind the breastbone |  |  |  |
| □Itchy eyes |  |  |  |
| □Tears |  |  |  |

1. ACT Total score:

| Q 1 | In the past 4 weeks, how much of the time did your asthma keep you from getting as much done at work, school or at home? | | | | | |
| --- | --- | --- | --- | --- | --- | --- |
|  | All of the time  1 | Most of the time  2 | Some of the time  3 | A little of the time  4 | None of the time  5 |  |
| Q 2 | During the past 4 weeks, how often have you had shortness of breath? | | | | | |
|  | More than once a day  1 | Once a day  2 | 3 to 6 times a week  3 | 1 to 2 times a week  4 | Not at all  5 |  |
| Q 3 | During the past 4 weeks, how often did your asthma symptoms (wheezing, coughing, shortness of breath, chest tightness or pain) wake you up at night or earlier than usual in the morning? | | | | | |
|  | 4 or more nights a week  1 | 2 to 3 nights a week  2 | Once a week  3 | Once or twice  4 | Not at all  5 |  |
| Q 4 | During the past 4 weeks, how often have you used your rescue inhaler or nebulizer medication (such as albuterol)? | | | | | |
|  | 3 or more times per day  1 | 1 or 2 times per day  2 | 2 or 3 times per week  3 | Once a week or less  4 | Not at all  5 |  |
| Q 5 | How would you rate your asthma control during the past 4 weeks? | | | | | |
|  | Not controlled at all  1 | Poorly controlled  2 | Somewhat controlled  3 | Well controlled  4 | Completely controlled  5 |  |

1. Leicester Cough Questionnaire (LCQ)

|  | 1 | 2 | 3 | 4 | 5 | 6 | 7 | Score |
| --- | --- | --- | --- | --- | --- | --- | --- | --- |
| 1.In the last 2 weeks, have you had chest or stomach pains as a result of your cough? | All of the time | Most of the time | A good bit of the time | Some of the time | A little of the time | Hardly any of the time | None of the time |  |
| In the last 2 weeks, have you been bothered by sputum (phlegm) production when you cough? | Every time | Most times | Several times | Some times | Occasionally | Rarely | Never |  |
| 3.In the last 2 weeks, have you been tired because of your cough? | All of the time | Most of the time | A good bit of the time | Some of the time | A little of the time | Hardly any of the time | None of the time |  |
| 4.In the last 2 weeks, have you felt in control of your cough? | None of the time | Hardly any of the time | A little of the time | Some of the time | A good bit of the time | Most of the time | All of the time |  |
| 5.How often during the last 2 weeks have you felt embarrassed by your coughing? | All of the time | Most of the time | A good bit of the time | Some of the time | A little of the time | Hardly any of the time | None of the time |  |
| 6.In the last 2 weeks, my cough has made me feel anxious | All of the time | Most of the time | A good bit of the time | Some of the time | A little of the time | Hardly any of the time | None of the time |  |
| 7.In the last 2 weeks, my cough has interfered with my job, or other daily tasks | All of the time | Most of the time | A good bit of the time | Some of the time | A little of the time | Hardly any of the time | None of the time |  |
| 8.In the last 2 weeks, I felt that my cough interfered with the overall enjoyment of my life | All of the time | Most of the time | A good bit of the time | Some of the time | A little of the time | Hardly any of the time | None of the time |  |
| 9.In the last 2 weeks, exposure to paints or fumes has made me cough | All of the time | Most of the time | A good bit of the time | Some of the time | A little of the time | Hardly any of the time | None of the time |  |
| 10.In the last 2 weeks, has your cough disturbed your sleep? | All of the time | Most of the time | A good bit of the time | Some of the time | A little of the time | Hardly any of the time | None of the time |  |
| 11.In the last 2 weeks, how many times a day have you had coughing bouts? | All of the time  (continuously) | Most times during  the day | Several times during  the day | Some times during  the day | Occasionally through  the day | Rarely | None |  |
| 12.In the last 2 weeks, my cough has made me feel frustrated | All of the time | Most of the time | A good bit of the time | Some of the time | A little of the time | Hardly any of the time | None of the time |  |
| 13.In the last 2 weeks, my cough has made me feel fed up | All of the time | Most of the time | A good bit of the time | Some of the time | A little of the time | Hardly any of the time | None of the time |  |
| 14.In the last 2 weeks, have you suffered from a hoarse voice as a result of your cough? | All of the time | Most of the time | A good bit of the time | Some of the time | A little of the time | Hardly any of the time | None of the time |  |
| 15.In the last 2 weeks, have you had a lot of energy? | None of the time | Hardly any of the time | A little of the time | Some of the time | A good bit of the time | Most of the time | All of the time |  |
| 16.In the last 2 weeks, have you worried that your cough may indicate serious illness? | All of the time | Most of the time | A good bit of the time | Some of the time | A little of the time | Hardly any of the time | None of the time |  |
| 17.In the last 2 weeks, have you been concerned that other people think something is wrong with you, because of your cough? | All of the time | Most of the time | A good bit of the time | Some of the time | A little of the time | Hardly any of the time | None of the time |  |
| 18.In the last 2 weeks, my cough has interrupted conversation or telephone calls | Every time | Most times | A good bit of the time | Some of the time | A little of the time | Hardly any of the time | None of the time |  |
| 19.In the last 2 weeks, I feel that my cough has annoyed my partner, family or friends | Every time I cough | Most times when  I cough | Several times when  I cough | Some times when  I cough | Occasionally when  I cough | Rarely | Never |  |

1. Previous diagnosis

□Asthma □ Cough variant asthma □ Chronic obstructive pulmonary diseases □Bronchiectasis □Gastroesophageal reflux disease □Allergic rhinitis □Sinusitis

1. Do you currently receive standardized treatment (ICS/ICS+LABA) for asthma for more than 3 months?

□Yes □No

1. Your current treatment level (GINA guideline) is:

□ Level 1 □ Level 2 □ Level 3 □ Level 4 □ Level 5

1. Was the cough symptom relieved after standard treatment?

□Totally relief □Partial relief □Not relief

1. Was the symptoms of wheezing and dyspnea relieved after standard treatment?

□Totally relief □Partial relief □Not relief

1. Lung function test

Pulmonary ventilation: Date: Height____cm; Weight____kg

FEV1__________L; FEV1/pred%__________

FVC __________L; FVC/pred%__________

FEV1/FVC__________%

MMEF: __________ L/sec; MMEF/pred%__________

PEF:__________ L/sec; PEF/pred%__________

FEF50:__________ L/sec; FEF50/pred%__________

FEF75:__________ L/sec; FEF50/pred%__________

Bronchodilation test □Negative □Positive

Post-diastolic FEV1_______L, Increase than base_______L, Improvement rate_______%

Bronchial provocation test □Negative □Positive

Stimulant: □Histamine □Methacholine

FEV1 after inhaling normal saline: _______L: FEV1/pred%__________

The cumulative dose of stimulant at the end of the challenge test is___μg (mg) FEV1:_____L

PD20: ________μg (mg)

1. Blood routine test, Date:

WBC:________10^9^/L Eos:________10^9^/L, _______%

1. Blood immunological test, Date:

T-IgE:_________kU/L S-IgE: □Negative □Positive

1. Induced sputum examination, Date:

Total number of cells: _____10^6^/g; N_____%; M_____%; L_____%; E_____%

1. Capsaicin cough sensitivity test, Date:

C2:_________μmol; C5:_________μmol

1. FeNO, Date:

_________ppb

1. Asthma control level classification: □Control □Partial control □Not control
2. Diagnosis: □ CVA □ CPA □ CA
