# Supplementary material for: Characteristics of different asthma phenotypes associated with cough: a prospective, multicenter survey in China
Source: Respir Res. 2022 Sep 12;23:243. doi: 10.1186/s12931-022-02104-8 (PMC9469623; doi:10.1186/s12931-022-02104-8)
Supplement: Supplementary file 3 — Additional file 3. Questions for indicating diagnosis of CVA, CPA and CA from questionnaire. [file 12931_2022_2104_MOESM3_ESM.docx]

**Supplement 3**

**Questions for indicating diagnosis of CVA, CPA and CA from questionnaire**

CVA: Cough variant asthma; CPA: Cough-predominant asthma; CA: Classic asthma.

Question 1: Since the onset of illness, did you have the following symptoms (choose one or more)?

□ Wheeze □ Dyspnea □Cough □Chest tightness

Question 2: Since the onset of illness, what were the predominant symptoms (choose one or more)?

□Wheeze □ Dyspnea □Cough □Chest tightness

Question 3: Since the onset of illness, what were the most predominant symptoms that troubled you (choose one)?

□Wheeze □ Dyspnea □Cough □Chest tightness

Question 4: Since the onset of illness, did you have cough as the main symptom lasting for more than 8 weeks?

□Yes, at present □Yes, in the past □No
