# Supplementary material for: Characteristics of different asthma phenotypes associated with cough: a prospective, multicenter survey in China
Source: Respir Res. 2022 Sep 12;23:243. doi: 10.1186/s12931-022-02104-8 (PMC9469623; doi:10.1186/s12931-022-02104-8)
Supplement: Supplementary file 4 — Additional file 4. Comparison of clinical characteristics among CVA, CPA and CA patients with regular and without regular treatment. [file 12931_2022_2104_MOESM4_ESM.docx]

**Supplement 4**

Comparison of clinical characteristics among CVA, CPA and CA patients with regular and without regular treatment.

|  | CVA | | | CPA | | | CA | | |
| --- | --- | --- | --- | --- | --- | --- | --- | --- | --- |
|  | With regular treatment | Without regular treatment | *P* | With regular treatment | Without regular treatment | *P* | With regular treatment | Without regular treatment | *P* |
| Number | 34 | 252 | NS | 274 | 723 | NS | 241 | 450 | NS |
| Age (years) | 44.1±16.9 | 42.5±15.5 | 0.481 | 48.1±14.9 | 45.0±14.1^^^ | 0.003 | 47.6±15.1 | 45.8±13.6^^^ | 0.201 |
| Asthmatic duration, month | 12.0 (6.0, 36.0)^*#^ | 8.8 (3.0, 34.5)^∫†^ | 0.095 | 40.0 (12.0, 103.5)^&#^ | 24.0 (6.0, 72.0)^^†^ | <0.001 | 71.0 (24.0, 180.0)^&*^ | 36.0 (12.0, 120.0)^^∫^ | <0.001 |
| ACT | 20 (17, 23) | 21 (18, 22) | 0.942 | 19 (16, 21) | 18 (15, 21)^^^ | <0.001 | 19 (16, 22) | 18 (14, 20)^^^ | <0.001 |
| FEV1%pred | 93.4±17.9^*#^ | 93.6±11.5^∫†^ | 0.548 | 75.3±22.6 | 80.3±19.6 | 0.002 | 70.6±21.5 | 77.9±19.5 | <0.001 |
| LCQ | 12.9 (9.4, 15.8) | 14.3 (11.8, 17.2) | 0.101 | 15.0 (11.6, 17.9) | 13.4 (10.7, 16.3) | <0.001 | 18.3 (13.8, 21.0)^&*^ | 18.9 (15.3, 21.0) ^^∫^ | 0.144 |
| Cough VAS | 55 (30, 70) | 50 (40, 65) | 0.938 | 40 (30, 60) | 50 (40, 70) | <0.001 | 20 (0, 30)^&*^ | 10 (0, 30) ^^∫^ | 0.038 |
| Induced sputum test (n) | 10 | 108 | NS | 63 | 232 | NS | 60 | 87 | NS |
| Sputum Eos% | 8.5 (0.2, 24.4) | 5.5 (1.0, 26.1) | 0.795 | 10.1 (1.0, 35.1) | 7.0 (1.0, 32.7) | 0.068 | 7.7 (0.7, 31.8) | 7.2 (1.0, 27.8) | 0.977 |
| Sputum Neu% | 74.3 (44.5, 79.3) | 55.2 (29.3, 80.9) | 0.386 | 57.5 (32.5, 78.0) | 60.0 (32.5, 85.0) | 0.593 | 60.1 (24.9, 82.5) | 62.2 (43.5, 86.0) | 0.245 |
| FeNO measurement (n) | 15 | 137 | NS | 91 | 306 | NS | 68 | 159 | NS |
| FeNO (ppb) | 28.0 (13.0, 62.0) | 37.0 (18.0, 78.0) | 0.465 | 42.0 (28.0, 90.0) | 39.5 (18.8, 76.5) | 0.059 | 35.0 (18.5, 69.3) | 43.0 (18.0, 71.0) | 0.489 |
| capsaicin cough challenge (n) | 5 | 80 | NS | 36 | 82 | NS | 30 | 28 | NS |
| LgC5 | 2.7 (1.2, 3.0) | 1.5 (0.3, 2.4) | 0.178 | 1.2 (0.3, 2.4)^#^ | 2.1 (1.2, 2.4) | 0.082 | 2.8 (1.7, 3.0) | 2.4 (1.3, 3.0) | 0.297 |

Data were presented as median (IQR) or mean ± SD. FEV1% pred: forced expiratory volume in 1 second in % predicted; ACT: asthma control test; LCQ: Leicester cough questionnaire; VAS: visual analogue scale; Eos: eosinophil; Neu: neutrophil; FeNO: fractional exhaled nitric oxide; LgC5: The lowest concentrations of capsaicin (C5) which evoked five or more coughs were obtained and the level of CRS was presented as the logarithm of C5 (LogC5). CVA: cough variant asthma; CPA: cough predominant asthma; CA: classic asthma. Regular treatment: inhaled corticosteroids (ICS)/ICS + long-acting Beta2-agonists (LABA). The patients were divided into two groups according to receiving regular treatment or not in the past 3 months. For patients with regular treatment: &: Compared to CVA, p<0.05; *: Compared to CPA, p<0.05; #: Compared to CA, p<0.05. For patients without regular treatment: ^: Compared to CVA, p<0.05; ∫: Compared to CPA, p<0.05; †: Compared to CA, p<0.05.
